# Sex differences in the lung ACE/ACE2 balance in hypertensive rats

**DOI:** 10.1042/BSR20211201

**Published:** 2021-12-07

**Authors:** Flavia L. Martins, Caio A.M. Tavares, Pamella A. Malagrino, Thiago Rentz, Acaris Benetti, Thiago M.S. Rios, Gabriel M.D. Pereira, Bruno Caramelli, Samantha K. Teixeira, José E. Krieger, Adriana C.C. Girardi

**Affiliations:** 1 Laboratory of Genetics and Molecular Cardiology, Heart Institute (InCor) – University of São Paulo Medical School, São Paulo, SP, Brazil; 2 Cardiogeriatric Unit, Heart Institute (InCor) – University of São Paulo Medical School, São Paulo, SP, Brazil; 3 Cardiology Interdisciplinary Medicine Unit, Heart Institute (InCor) – University of São Paulo Medical School, São Paulo, SP, Brazil

**Keywords:** angiotensin II, angiotensin-(1-7), Genotype-Tissue Expression project, hypertension, sexual dimorphism

## Abstract

The angiotensin-converting enzyme (ACE)/Angiotensin II (Ang II) and angiotensin-converting enzyme 2 (ACE2)/angiotensin-(1-7) (Ang-(1-7)) pathways are coexpressed in most tissues. The balance between these pathways determines, at least in part, whether tissue damage will occur in response to pathological stimuli. The present study tested the hypothesis that male sex and high blood pressure are associated with ACE/ACE2 imbalance in the lungs. Experiments were conducted in male and female Wistar rats and spontaneously hypertensive rats (SHRs). Lung ACE and ACE2 gene expression was also evaluated in normotensive and hypertensive humans using the Genotype-Tissue Expression (GTEx) project. Compared with Wistar rats and female SHRs, male SHRs displayed reduced lung ACE2 mRNA, ACE2 protein abundance and ACE2 activity, and increased Ang II concentration. Lung ACE mRNA levels were higher in male SHRs than in Wistar rats, whereas lung ACE protein abundance and activity were similar among the four groups of rats. Lung Ang-(1-7) concentration was higher in female than in male SHRs (89 ± 17 vs. 43 ± 2 pg/g, *P*<0.05). Lung ACE to ACE2 mRNA expression in hypertensive patients was significantly higher than that in normotensive subjects. Taken together, these results demonstrate that male hypertensive rats display imbalance between the ACE/Ang II and ACE2/Ang-(1-7) pathways in the lungs mainly attributable to ACE2 down-regulation. Further studies should be conducted to investigate whether this imbalance between ACE/ACE2 may promote and accelerate lung injury in respiratory infections, including coronavirus disease 2019 (COVID-19).

## Introduction

Biological sex is recognized as a crucial determinant of susceptibility and clinical outcomes in non-communicable and infectious diseases. Sex differences have been well investigated in cardiology since several epidemiological studies demonstrated that premenopausal women are less prone to developing cardiovascular disease (CVDs) than age-matched men [1–3]. In accordance, high blood pressure, a major risk factor for CVDs, prevails in men before the age of 50 [4–6]. Similarly, male bias has emerged in the coronavirus disease 2019 (COVID-19) pandemic, caused by severe acute respiratory syndrome coronavirus 2 (SARS-CoV-2). Sex differences have been consistently implicated in the severity of COVID-19 [7], including mortality and the need for admission to intensive care [8]. One potential mechanism responsible for sex dimorphism in hypertension and COVID-19 is the effect of sex steroid hormones on the renin–angiotensin system (RAS) [9,10].

Abnormal activation of the classical RAS components, ultimately leading to the up-regulation of angiotensin II (Ang II) and activation of the Ang II type 1 receptor (AT1R), contributes to the development and progression of hypertension [11–15]. Conversely, angiotensin-converting enzyme 2 (ACE2) functions as a counterregulatory enzyme that cleaves Ang II to form angiotensin-(1-7) (Ang-(1-7)), which in turn activates the Mas receptor (MasR). The ACE2/Ang-(1-7) pathway exerts a protective role against hypertension by reducing blood pressure, promoting vasodilation, increasing the renal excretion of sodium and water, and exerting anti-inflammatory and antioxidant effects [16]. Interestingly, renal ACE2 activity and expression were reduced in ovariectomized hypertensive rats, whereas administration of 17-β estradiol restored ACE2 levels and conferred renoprotection to these animals [17]. These findings raise the possibility that estradiol-replete women may be protected from hypertensive renal disease at least partly by estradiol-mediated up-regulation of ACE2.

ACE2 is also the functional receptor for SARS-CoV-2 host cell entry [18], thus providing a biological link among hypertension, biological sex, and COVID-19. Loss of the ACE2 protective arm in the lungs may create an imbalance that favors the classic RAS, unleashing a cascade of harmful effects that leads to severe acute respiratory failure in COVID-19 [19–23]. Indeed, a causal relationship between imbalance of the angiotensin-converting enzyme (ACE)/ACE2 axis and acute respiratory distress syndrome has been established through the use of genetically modified animals, demonstrating that the ACE/Ang II/AT1R axis drives severe acute lung failure, whereas ACE2 protects against it [24]. Sex modulation of the lung ACE to ACE2 ratio in systemic arterial hypertension, however, remains elusive. The present study was undertaken to test the hypothesis that male sex and high blood pressure are associated with ACE/ACE2 imbalance in the lungs. To this end, we investigated the sex-specific modulation of ACE and ACE2 at the transcriptional, translational, and post-translational levels in the lungs of normotensive and hypertensive rats. Lung ACE to ACE2 gene expression was further evaluated in hypertensive and normotensive humans using the Genotype-Tissue Expression (GTEx) project.

## Methods

### Animals

All experiments were carried out following the ethical principles of animal research of the Brazilian College of Animal Experimentation and had been approved by the Institutional Animal Care and Use Committee (protocol number #1550/2020). All animal experiments took place in the Heart Institute (InCor), University of São Paulo Medical School, São Paulo, SP, Brazil. Blood pressure in conscious, restrained rats was measured noninvasively by tail-cuff plethysmography (BP-2000 Blood Pressure Analysis System, Visitech Systems, Apex, NC) 1 day before euthanasia as previously described [25]. Experiments were performed using 12-week-old male (*n*=10, BP = 129 ± 1 mmHg) and female (*n*=10, BP = 118 ± 1 mmHg) Wistar rats and age-matched male (*n*=9, BP = 185 ± 3 mmHg) and female (*n*=8, BP = 161 ± 2 mmHg) spontaneously hypertensive rats (SHRs) (Supplementary Figure S1). Rats were housed at the Heart Institute (InCor) animal facility under a 12-h dark–light cycle. Access to food and water was provided *ad libitum*. The rats were anesthetized intraperitoneally with pentobarbital (50 mg/kg body weight), terminal arterial blood samples were collected from the abdominal aorta to obtain serum, and the lungs were rapidly removed. Anesthetized rats were killed by decapitation.

### Quantitative reverse transcription-polymerase chain reaction

Total RNA was isolated from lung tissue using TRIzol reagent (Thermo Fisher Scientific, Carlsbad, CA). The Super-Script IV Reverse Transcriptase (Thermo Fisher Scientific) was used to synthesize lung first-strand cDNA. Gene expression was determined by quantitative reverse transcription-polymerase chain reaction (RT-PCR). Real-time RT-PCRs were performed using SYBR Green PCR Master Mix-PE (Applied Biosystems, Foster City, CA) in the ABI Prism 7700 Sequence Detection System (Applied Biosystems). The following oligonucleotide primers were used: ACE, CCAGAGGGAATTGACCTAGAGAC (forward) and ACACTTCCTTGTTTTTCTGAAGCA (reverse); ACE2, GAGCCCATATGCCGACCAAA (forward) and CTCACCCATACGTCTGCCTC (reverse); and cyclophilin, AATGCTGGACCAAACACAAA (forward) and CCTTCTTTCACCTTCCCAAA (reverse). All samples were assayed in triplicate. The relative expression was normalized to that of cyclophilin.

### Lung homogenate and membrane protein isolation

The lungs were homogenized in ice-cold PBS buffer (150 mM sodium chloride, 2.8 mM monobasic sodium phosphate, 7.2 mM dibasic sodium phosphate, pH 7.4) containing protease (1 μM pepstatin A, 1 μM leupeptin, 230 μM phenylmethylsulfonyl fluoride (PMSF)) (Sigma–Aldrich) and phosphatase inhibitors (15 mM sodium fluoride and 50 mM sodium pyrophosphate) (Sigma–Aldrich) using a Potter–Elvehjem-style tissue grinder (POLYMIX PX-SR50E, Kinematica, Luzern, Switzerland). The homogenate was centrifuged at 2000×***g*** for 10 min at 4°C. Part of the supernatant was stored at −80°C, and the other part was centrifuged at 100000×***g*** for 1 hour at 4°C. The pellet was then resuspended in fresh PBS with protease and phosphatase inhibitors and stored at −80°C. The determination of protein concentration in the samples was by the Lowry method [26].

### SDS/PAGE and immunoblotting

Equal protein amounts of lung membrane proteins were run on an SDS/PAGE polyacrylamide gel (7.5%) and transferred to a polyvinylidene difluoride (PVDF) membrane (Immobilon-P, Merck Millipore, Darmstadt, Germany) at 350 mA for 8–10 h at 4°C. Then, the PVDF membranes were incubated with blocking solution (5% nonfat dry milk or 5% bovine serum albumin and 0.1% Tween 20 in PBS, pH 7.4) for 1 h and overnight (4°C) with specific primary antibodies: a rabbit monoclonal antibody against ACE (1:1000; ab254222, Abcam, Cambridge, MA), a rabbit polyclonal antibody against ACE2 (1:1000; ab15348, Abcam) or a mouse monoclonal antibody against β-actin (1:5000; ab6276, Abcam). Proteins were detected using horseradish peroxidase-conjugated secondary antibodies (1:2000; Jackson ImmunoResearch, West Grove, PA). The bound antibodies were detected using an enhanced chemiluminescence system (GE Healthcare, United Kingdom) according to the manufacturer’s protocols. The visualized bands were digitized using an Image Scanner (GE HealthCare) and quantified using the Scion Image Software package (Scion Corporation, Frederick, MD).

### Lung ACE and ACE2 activity

ACE and ACE2 activity from lung membrane proteins (2 µg for ACE and 20 µg for ACE2) were determined using the ACE activity assay kit (fluorometric) from Sigma–Aldrich (CS0002, St. Louis, MO) and the ACE2 activity assay kit (fluorimetric) from Abcam (ab273373), according to the manufacturer’s instructions. Both assays are based on the cleavage of synthetic fluorogenic peptides and were conducted in the presence or absence of selective inhibitors of ACE (10 µM captopril) and ACE2 (20 µM). Fluorescence was measured at 320 nm excitation and 420 nm emission wavelength in kinetic mode for 1 h at 37°C (Molecular Devices, San José, CA, U.S.A.). All samples and standards were assayed in duplicate. Enzyme activity was expressed as milliunits (mU) per mg, i.e., the amount of enzyme that releases 1 pmol of fluorescent product from the substrate, in 1 min, under the conditions of the assay method.

### Lung ADAM17 activity

A disintegrin and metalloprotease 17 (ADAM17) activity was measured using a fluorescence-quenched ADAMs substrate (Mca–Pro–Leu–Ala–Gln–Ala–Val–Dpa–Arg–Ser–Ser–Ser–Arg–NH_2_ (R&D Systems). Briefly, 5 µg of lung membrane proteins were incubated with reaction buffer (50 mM acetic acid, pH 4.5, 100 mM sodium chloride) in the presence or absence of the ADAM17 inhibitor TAPI 0 (10 µM; Tocris Biosciences, Minneapolis, MN, U.S.A.). Samples were incubated with 10 µM of the fluorescent substrate in reaction buffer (final volume of 200 µl) at 37°C for 5 h. Enzymatic activity was continuously monitored with SpectraMax M5 (Molecular Devices, San José, CA) by measuring fluorescence at 320 nm excitation and 420 nm emission wavelength. Experiments were carried out in duplicate. Results were expressed as relative fluorescence units (RFUs/µg/h).

### Lung Ang II and Ang-(1-7) concentration

Enzyme-linked immunosorbent assay (ELISA) was employed to measure the concentration of Ang II (EKC38669, Biomatik, Cambridge, ON) and Ang-(1-7) (EKC38664, Biomatik) in lung homogenates containing protease inhibitors, according to the manufacturer’s instructions.

### Serum ACE and ACE2 concentration

The arterial blood was collected into vacuum tubes with a gel separator (Vacutainer SST Advance, Franklin Lakes, NJ) and centrifuged at 1200×***g*** for 10 min at 4°C to obtain serum. The serum concentrations of ACE and ACE2 were determined by ELISA (Biomatik). Experiments were conducted according to the manufacturers’ instructions.

### Human gene expression data

Lung ACE and ACE2 gene expression analysis in male (*n*=94) and female (*n*=18) hypertensive and male (*n*=216) and female (*n*=56) normotensive subjects was performed using the GTEx database (https://www.gtexportal.org/home/). Detailed information regarding tissue and patient collection, as well as gene expression experiments, are described elsewhere [27]. Briefly, paired-end RNA-seq was generated using Illumina TruSeq Protocol and aligned to the human genome (GRCh38/hg38) using STAR v2.5.3a, based on the GENCODE v26 annotation (https://www.gencodegenes.org/human/release_26.html). Gene-level quantification was estimated as transcripts per million using RNA-SeQC v1.1.9 [28]. Patient information was obtained upon request for controlled-access data (dbGaP accession phs000424.v8.p2 #90490-2) [29]. Briefly, patients’ information was extracted from medical reports obtained from deceased donors’ next-of-kin. Thus, information about medical history and the use of medications is not specific. We considered as hypertensive those individuals who displayed the following terms in their medical history: hypertension, essential hypertension, high blood pressure, or medication for hypertension, including nicardipine, enalapril, hydrochlorothiazide, bisoprolol, and clevidipine. Patients’ systolic and diastolic blood pressure were not available since samples were collected postmortem.

### Statistical analyses

The experimental animal results are reported as the mean ± standard error of the mean (SEM). The normality of the distribution of all variables was evaluated using the Kolmogorov–Smirnov test. If the numerical data were normally distributed, comparisons among the means were carried out using two-way analysis of variance (ANOVA) followed by Tukey’s post hoc test. Otherwise, the nonparametric Kruskal–Wallis test was used. The Pearson correlation test was used to measure the strength of associations between two parametric variables, and the Spearman correlation test was employed to assess the relationships between nonparametric variables. Differences for which *P*<0.05 were considered significant. Statistical analyses were performed using GraphPad Prism 8 software (GraphPad Software, La Jolla, CA). Differential gene expression in the human lung was assessed using the DESeq2 R package from Bioconductor, which employs a negative binomial distribution to estimate dispersion and model differential expression (http://bioconductor.org/packages/release/bioc/html/DESeq2.html). Differences in the ACE/ACE2 mRNA ratio between normotensive and hypertensive individuals were assessed using the generalized linear model with gamma distribution using transcripts per million normalized data using the glm function in R after outlier removal (GTEx_Analysis_2017-06-05_v8_RNASeQCv1.1.9_gene_tpm.gct).

## Results

### Lung ACE and ACE2 mRNA expression, protein abundance, and enzymatic activity in male and female normotensive and hypertensive rats

**Figure 1:**
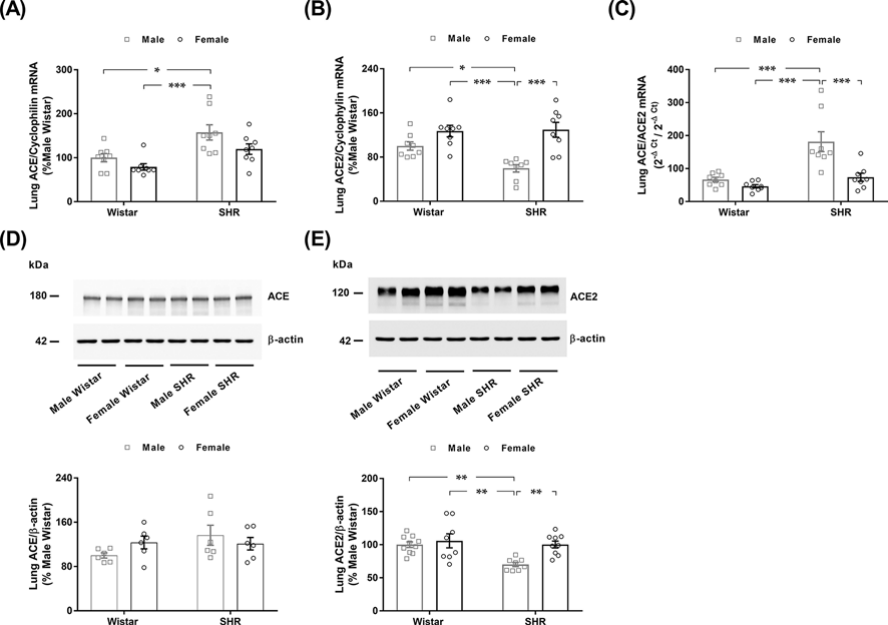
Lung ACE and ACE2 mRNA and protein expression in male and female normotensive (Wistar) and hypertensive (SHR) rats The gene expression of (**A**) ACE and (**B**) ACE2 was quantitatively measured by RT-PCR and normalized to cyclophilin and is expressed as the % of male Wistar rats. (**C**) Lung ACE/ACE2 gene expression ratio was expressed as 2^−Δ*C*_t_^/2^−Δ*C*_t_^. The protein expression of (**D**) ACE and (**E**) ACE2 was determined by immunoblotting of lung membrane proteins and normalized to β-actin and is expressed as the % of male Wistar rats (Supplementary Figures S3 and S4). The values represent individual measurements and the means ± SEMs. *P<0.05; **P<0.01; ***P<0.001.

Lung ACE mRNA expression was similar in males and females of both strains (Figure 1A). Conversely, male SHRs displayed higher lung ACE mRNA expression than male and female Wistar rats. No difference in the expression of ACE2 mRNA was found between male and female Wistar rats (Figure 1B). However, male SHRs exhibited lower lung ACE2 mRNA expression than female SHRs and male and female Wistar rats. Figure 1C shows that the ACE to ACE2 mRNA levels were remarkably higher in the lungs of male SHRs than in the lungs of the three other groups of animals (Figure 1C).

As seen in Figure 1D, the protein abundance of ACE did not vary among the four groups of rats. According to the mRNA data, male SHRs displayed an ∼40% decrease in the lung membrane protein abundance of ACE2 (Figure 1E). No difference in the lung membrane protein abundance of ACE2 was observed between male and female Wistar rats (Figure 1E). We could not determine the protein ratio of ACE to ACE2 in the lung, given the semi-quantitative nature of immunoblotting analysis and the different antibody characteristics for each protein.

**Figure 2:**
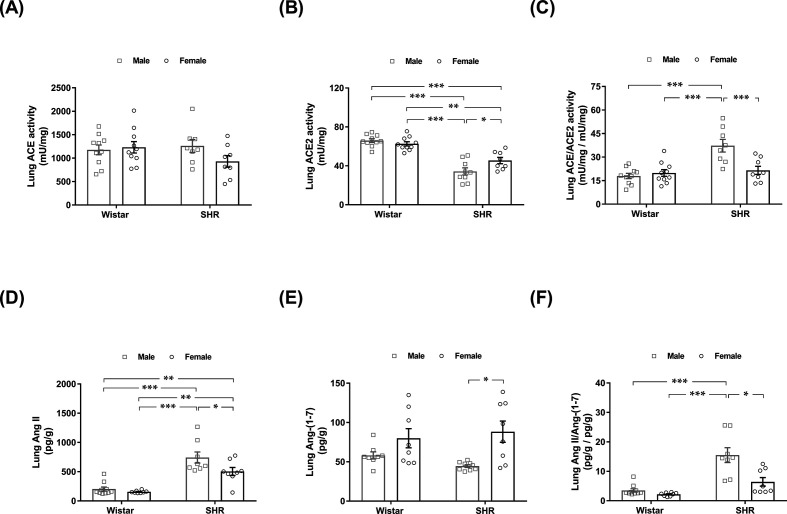
Lung activity of ACE and ACE2 and lung concentration of their respective products, Ang II and Ang-(1-7), in male and female normotensive (Wistar) and hypertensive (SHR) rats The activities of (**A**) ACE and (**B**) ACE2 were assayed by a fluorogenic method. (**C**) Lung ACE/ACE2 activity ratio. Lung (**D**) Ang II and (**E**) Ang-(1-7) concentrations were determined by ELISA. (**F**) Lung Ang II/Ang-(1-7) peptide concentration ratio. The values represent individual measurements and the means ± SEMs. *P<0.05; **P<0.01; ***P<0.001.

Figure 2A shows that ACE activity in the lungs was similar among the four groups of rats. Additionally, no difference in lung ACE2 activity was found between male and female Wistar rats (Figure 2B). In contrast, lung ACE2 activity was reduced in SHRs compared with Wistar rats, regardless of biological sex. In addition, lung ACE2 activity was decreased by 25% in male SHRs compared with female SHRs (*P*<0.05) (Figure 2B). As seen in Figure 2C, lung ACE to ACE2 activity was approximately two-fold higher in male SHRs than in Wistar rats and female SHRs.

### Ang II and Ang-(1-7) concentrations in the lungs of male and female normotensive and hypertensive rats

As shown in Figure 2D, the lung concentrations of Ang II in male and female normotensive rats were similar (203 ± 34 vs. 156 ± 8 pg/g). Both male and female hypertensive rats displayed increased Ang II levels than those in Wistar rats; however, this increase was most prominent in male SHRs (∼3-fold higher in male hypertensive rats vs. male normotensive animals and ∼4.5-fold higher than female Wistar rats). A significant difference in lung Ang II concentration was also found between male and female SHRs (743 ± 95 vs. 501 ± 68 pg/g, *P*<0.05). Biological sex did not affect the concentration of Ang-(1-7) in the lungs of Wistar rats (Figure 2E). Conversely, the lung content of Ang-(1-7) was lower in hypertensive males than in hypertensive females (43 ± 2 vs. 89 ± 17 pg/g, *P*<0.05). The lung ratios of Ang II to Ang-(1-7) in male and female Wistar rats were similar. In accordance with the enzymatic activity data (Figure 2C), the lung ratio of Ang II to Ang-(1-7) was remarkably increased in male SHRs compared with the other three groups of rats (Figure 2F).

### Serum concentrations of ACE and ACE2 in male and female normotensive rats

**Figure 3:**
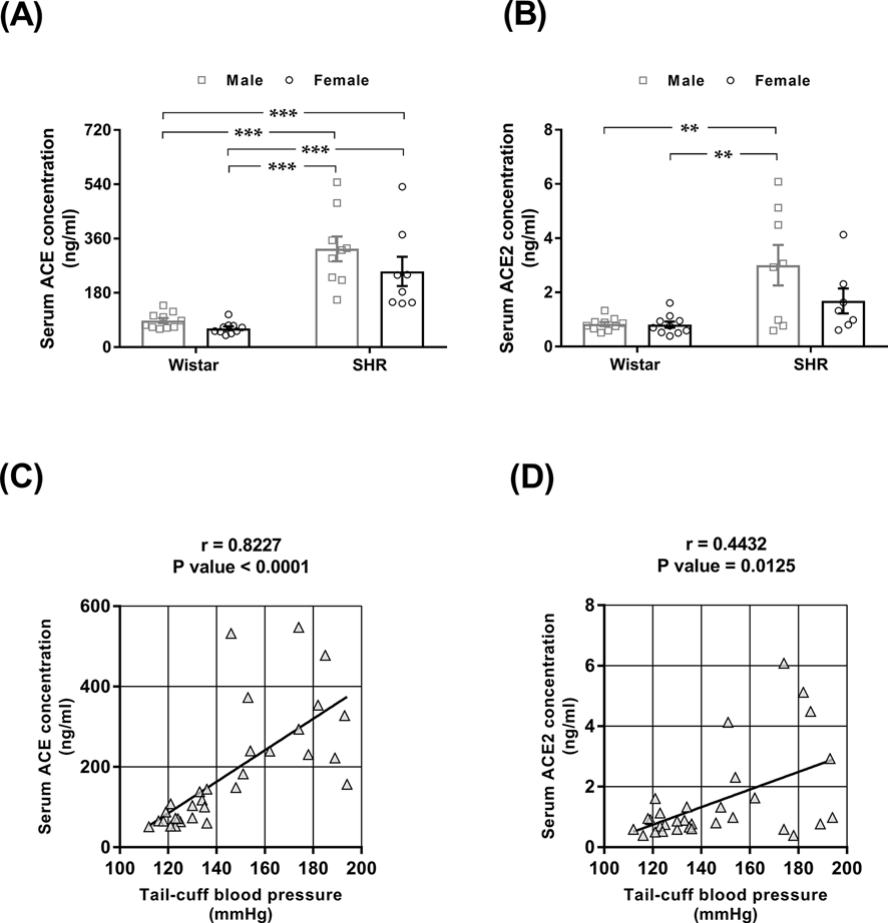
Serum ACE and ACE2 concentrations and their correlation with blood pressure in male and female normotensive (Wistar) and hypertensive (SHR) rats Serum (**A**) ACE and (**B**) ACE2 concentrations were determined by ELISA. The values represent individual measurements and the means ± SEMs. **P<0.01 and ***P<0.001. Correlations between the serum (**C**) ACE and (**D**) ACE2 concentrations and tail-cuff blood pressure. The correlation coefficients (*r*) and *P*-values were obtained by Pearson’s correlation test, and lines were obtained by linear regression plotting.

No difference in serum ACE concentration was noted between males and females of either strain (Figure 3A). SHRs exhibited a higher serum ACE concentration than normotensive rats, regardless of sex. The serum ACE2 concentration was increased in male SHRs compared with the three other groups of animals (Figure 3B). The relationships between blood pressure and ACE and ACE2 serum concentrations are shown in Figure 3C,D. As seen in Figure 3C,D, the higher the blood pressure was, the higher the serum concentrations of ACE (Figure 3C) and ACE2 were (Figure 3D).

### Lung ADAM17 activity in male and female normotensive and hypertensive rats

**Figure 4:**
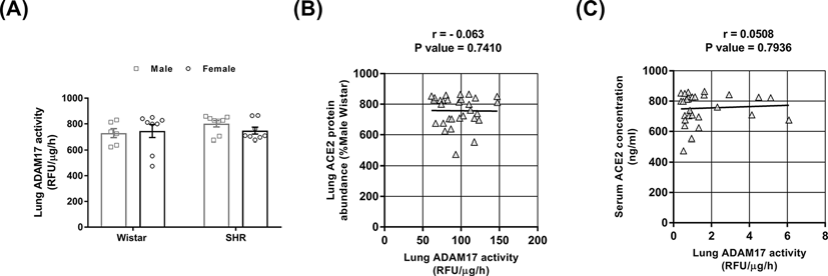
Lung ADAM17 activity and its correlation with lung ACE2 protein and serum abundance in male and female normotensive (Wistar) and hypertensive (SHR) rats (**A**) Lung ADAM17 activity was determined by a fluorescent enzymatic assay in the presence or absence of TAPI 0. The TAPI 0 sensitive enzymatic activity was expressed as RFUs/µg/h. The values represent individual measurements and the means ± SEMs. The absence of a correlation between lung ADAM17 activity and (**B**) lung ACE2 protein or (**C**) serum ACE2 concentration. The correlation coefficients (*r*) and *P*-values were obtained by Pearson’s correlation test, and lines were obtained by linear regression plotting.

To evaluate whether down-regulation of lung ACE2 in male SHRs could be at least partially attributed to ectodomain shedding, we measured the activity of ADAM17, a disintegrin, and metalloprotease that cleaves and releases the catalytically active ectodomain of ACE2 to the circulation [30]. The lung activity of ADAM17 was similar among the four groups of rats (Figure 4A). Accordingly, no significant correlation was found between ADAM17 activity and lung ACE2 protein abundance (Figure 4B) or serum ACE2 abundance (Figure 4C).

### Relationship between serum and the lung expression of ACE and ACE2

**Figure 5:**
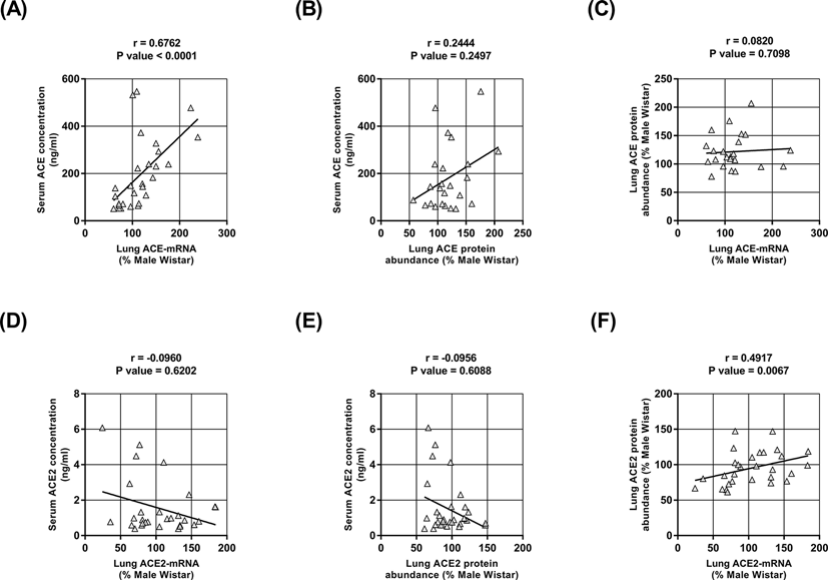
The relationship between the lung expression and serum concentrations of ACE and ACE2 (**A**) Correlation between serum ACE concentration and lung ACE mRNA level. (**B**) The absence of a correlation between serum ACE concentration and lung ACE protein abundance. (**C**) The absence of a correlation between lung ACE protein abundance and lung ACE mRNA level. (**D**) The absence of a correlation between serum ACE2 concentration and lung ACE2 mRNA level. (**E**) The absence of a correlation between serum ACE2 abundance and lung ACE2 protein abundance. (**F**) Correlation between lung ACE2 protein abundance and lung ACE2 mRNA level. The correlation coefficients (*r*) and *P*-values were obtained by Pearson’s correlation test, and lines were obtained by linear regression plotting.

The serum abundance of ACE significantly correlated with lung ACE mRNA level (Figure 5A) but not with ACE protein abundance (Figure 5B). Accordingly, no association between ACE mRNA and ACE protein abundance was found (Figure 5C). Serum ACE2 abundance did not correlate with either lung ACE2 mRNA level (Figure 5D) or ACE2 protein abundance (Figure 5E); however, lung ACE2 mRNA levels and ACE2 protein abundance were positively correlated (Figure 5F).

### Lung ACE and ACE2 mRNA expression in normotensive and hypertensive humans

**Figure 6:**
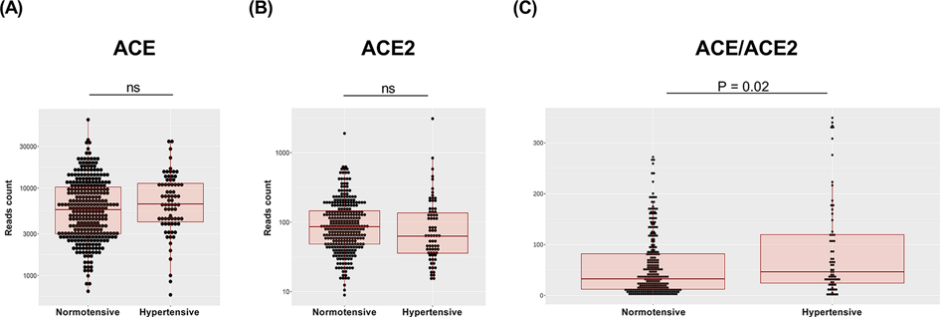
Lung ACE and ACE2 mRNA expression in normotensive and hypertensive patients Normalized (**A**) ACE mRNA and (**B**) ACE2 mRNA expression data and the (**C)** ACE to ACE2 mRNA ratio from normotensive and hypertensive patients whose lung samples were available in the GTEx database. Boxplots show the minimum, first quartile, median, third quartile, and maximum values. Each dot represents an individual data point.

Figure 6 presents data on lung ACE and ACE2 mRNA expression in hypertensive and normotensive subjects. No difference in lung ACE gene expression level was found between normotensive and hypertensive patients (log2 fold change = 0.169; *P*=0.22) (Figure 6A). A trend towards decreased lung ACE2 mRNA expression was observed in hypertensive patients (Figure 6B), but the difference did not reach statistical significance (log2 fold change = −0.168; *P*=0.08). However, as seen in Figure 6C, lung ACE to ACE2 mRNA expression in hypertensive patients was significantly higher than that in normotensive subjects.

## Discussion

The main findings of our study can be summarized as follows: (i) male hypertensive rats displayed imbalance between the ACE/Ang II and ACE2/Ang-(1-7) pathways in the lungs; (ii) this RAS imbalance resulted in a higher lung Ang II concentration that is mainly attributable to decreased lung ACE2 activity since neither high blood pressure nor biological sex was associated with changes in lung enzymatic ACE function; (iii) down-regulation of lung ACE2 activity in male hypertensive rats was accompanied by decreased ACE2 protein abundance and ACE2 mRNA expression but did not seem to be dependent on increased ADAM17-mediated shedding; (iv) the lung ACE/ACE2 mRNA ratio was increased in hypertensive patients, thereby suggesting that the RAS balance may be shifted towards the classical RAS in human subjects with high blood pressure.

The present study mainly focused on the relationships between biological sex, high blood pressure, and the balance between the RAS pathways in the lungs. However, it also provides interesting insights regarding sex dimorphism-mediated modulation of lung ACE2 in hypertension. Based on the results shown here, we suggest that down-regulation of ACE2 activity in the lungs leads to the decreased consumption of Ang II and that lower Ang-(1-7) production may be primarily due to decreased ACE2 mRNA transcription and translation. Indeed, a high degree of correlation was found between lung ACE2 mRNA and ACE2 protein abundance. The fact that the activity of the ACE2 sheddase ADAM17 [30] was similar among the four groups of rats demonstrates that neither biological sex nor blood pressure affects its enzymatic activity in the lungs. This finding also suggests that pulmonary plasma membrane ACE2 expression is not primarily regulated by ADAM17-induced post-translational modifications in SHRs. Accordingly, no correlation was found between lung ADAM17 activity and lung ACE2 protein abundance or serum concentration. In addition, no relationship was found between lung ACE2 expression and serum ACE2 concentration, thereby suggesting that the lungs are not the primary source of serum ACE2. It is worth mentioning that several cellular processes, such as protein degradation and post-translational modification mechanisms other than ADAM17-mediated shedding, might impact ACE2 activity in the lungs, but these processes were not evaluated in the present study.

High-serum ACE and ACE2 are known risk factors for CVD [31–35] and, as revealed more recently, COVID-19 severity [21,36]. Consistent with these studies, we found that rats with high blood pressure, regardless of biological sex, displayed higher serum ACE and ACE2 concentrations. In addition, a strong positive correlation between blood pressure and serum ACE levels was noted, and a moderate correlation between blood pressure and serum ACE2 content was noted. Because the circulating pool of ACE and ACE2 represents the sum of ectodomain shedding from the cell surface among a wide variety of tissues minus plasma clearance [37,38], the relative contributions from different tissues should be weighed based on the organ-specific gene expression of ACE/ACE2 and local shedding [39,40]. As mentioned above, our results suggest that in the setting of chronic hypertension, the contribution of lung ACE2 shedding to serum ACE2 is probably minor. However, chronic hypertension seems to influence lung ACE2 expression since a moderate correlation between blood pressure and lung ACE2 protein abundance has been found (Supplementary Figure S2). In contrast, high blood pressure is associated with both augmented lung ACE mRNA and ACE shedding from the cell surface, leading to increased serum ACE concentrations but not ACE cell surface lung protein abundance. These data also suggest that under not only physiological conditions [41] but also chronic hypertension, the lung is one of the primary sources of serum ACE.

The finding that ACE/ACE2 mRNA expression is elevated in the lungs of hypertensive patients compared with those of age-matched normotensive individuals provides biological plausibility that our experimental results in rats can be translated into humans. In addition, since our samples were from both male and presumably postmenopausal women in the majority (average age higher than 55 years, Supplementary Table S1), we can speculate that the RAS imbalance observed in hypertensive males may be primarily due to the influence of sex hormones. Nevertheless, we have to acknowledge as a limitation of our study that samples from females for gene expression analysis were underrepresented. Therefore, we could not reliably analyze the influence of biological sex, and no conclusions about the sex-biased lung ACE/ACE2 mRNA-expression ratio in hypertensive patients could be made. Future studies assessing the impact of biological sex and menopausal status on human lung tissues will add important information about the mechanisms regulating the ACE/ACE2 expression ratio and might reveal the exact role of sex hormones and sex chromosomes.

Over the last year, several reviews and opinion articles have defended the view that a disequilibrium between the classic RAS and its opposing protective arm in the lungs plays a crucial role in the pathophysiology of COVID-19 [19,22,23,42]. This study found that high blood pressure and male sex shift the RAS balance in the lungs towards the vasoconstrictive, profibrotic, and proinflammatory ACE/Ang II axis. Notably, during the early stages of the pandemic, concerns that increased ACE2 cell surface expression could be a causal factor for COVID-19 severity due to augmented viral cell invasion and replication were raised. Some authors even postulated that hypertensive patients treated with angiotensin II type 1 receptor blockers (ARBs) or angiotensin-converting enzyme inhibitors (ACEis), which up-regulate ACE2, could be at higher risk for increased COVID-19 severity, and replacement with alternative pharmacological treatments was advocated [43–45]. However, data from observational studies and randomized clinical trials have consistently contradicted this hypothesis by demonstrating a lack of an association between the chronic use of either ACEis or ARBs and increased disease severity [46–48] or susceptibility to COVID-19 [49]. Indeed, a large observational study found a lower COVID-19 risk in hypertensive patients treated over a long period with ACEis or ARBs than with other antihypertensive drugs [50], suggesting that RAS inhibitors may, in fact, exert a protective effect. The data showed herein strengthen the notion that future research should consider the impact of sex on RAS dysregulation in COVID-19 patients, as sex-biased differences due to hypertension or a class of antihypertensive medications might occur.

To the best of our knowledge, this is the first study to demonstrate sexual dimorphism in lung ACE2 regulation at the transcriptional and translational levels in hypertensive rats. The combination of down-regulated lung ACE2 and unchanged ACE protein abundance and activity shifts the lung RAS balance towards the proinflammatory ACE/Ang II axis. Additionally, gene expression analysis in human lung samples showed higher ACE/ACE2 in hypertensive patients, suggesting that our findings in experimental animals can potentially be extrapolated to humans. Further research is needed to address whether the use of RAS inhibitors, including AT1R receptor blockers and ACEis, which up-regulate the ACE2/Ang-(1-7)/MasR axis, may help to mitigate the effects of COVID-19 in male and possibly postmenopausal female hypertensive patients.

## Supplementary Material

Supplementary Figures S1-S4 and Table S1

## Data Availability

The raw data supporting the conclusions of this article will be made available by authors without undue reservation.

## Abbreviations

ACE: angiotensin-converting enzyme
ACE2: angiotensin-converting enzyme 2
ACEi: angiotensin-converting enzyme inhibitor
ADAM17: a disintegrin and metalloprotease 17
Ang II: angiotensin II
Ang-(1-7): angiotensin-(1-7)
ARB: angiotensin II type 1 receptor blocker
AT1R: angiotensin II type 1 receptor
COVID-19: coronavirus disease 2019
CVD: cardiovascular disease
ELISA: enzyme-linked immunosorbent assay
GTEx: Genotype-Tissue Expression
MasR: Mas receptor
PVDF: polyvinylidene difluoride
RAS: renin–angiotensin system
RT-PCR: reverse transcription-polymerase chain reaction
SARS-CoV-2: severe acute respiratory syndrome coronavirus 2
SHR: spontaneously hypertensive rat
