# Supplementary material for: Sex differences in the lung ACE/ACE2 balance in hypertensive rats
Source: Biosci Rep. 2021 Dec 7;41(12):BSR20211201. doi: 10.1042/BSR20211201 (PMC8655502; doi:10.1042/BSR20211201)
Supplement: Supplementary Figures S1-S4 and Table S1 [file BSR-2021-1201_supp.pdf]

SUPPLEMENTAL MATERIAL

Sex differences in the lung ACE/ACE2 balance in hypertensive rats

Flavia L. Martins[1]*, Caio A. M. Tavares[2]*, Pamella A. Malagrino[1], Thiago Rentz[1], Acaris Benetti[1], Thiago M. S. Rios[1], Gabriel M. D. Pereira[1], Bruno Caramelli[3], Samantha K. Teixeira[1], José E. Krieger[1], Adriana C. C. Girardi[1]

[1]Laboratory of Genetics and Molecular Cardiology, [2]Cardiogeriatric Unit, [3]Cardiology Interdisciplinary Medicine Unit, Heart Institute (InCor) – University of São Paulo Medical School, São Paulo, SP, Brazil

* contributed equally to this work

Figure S1

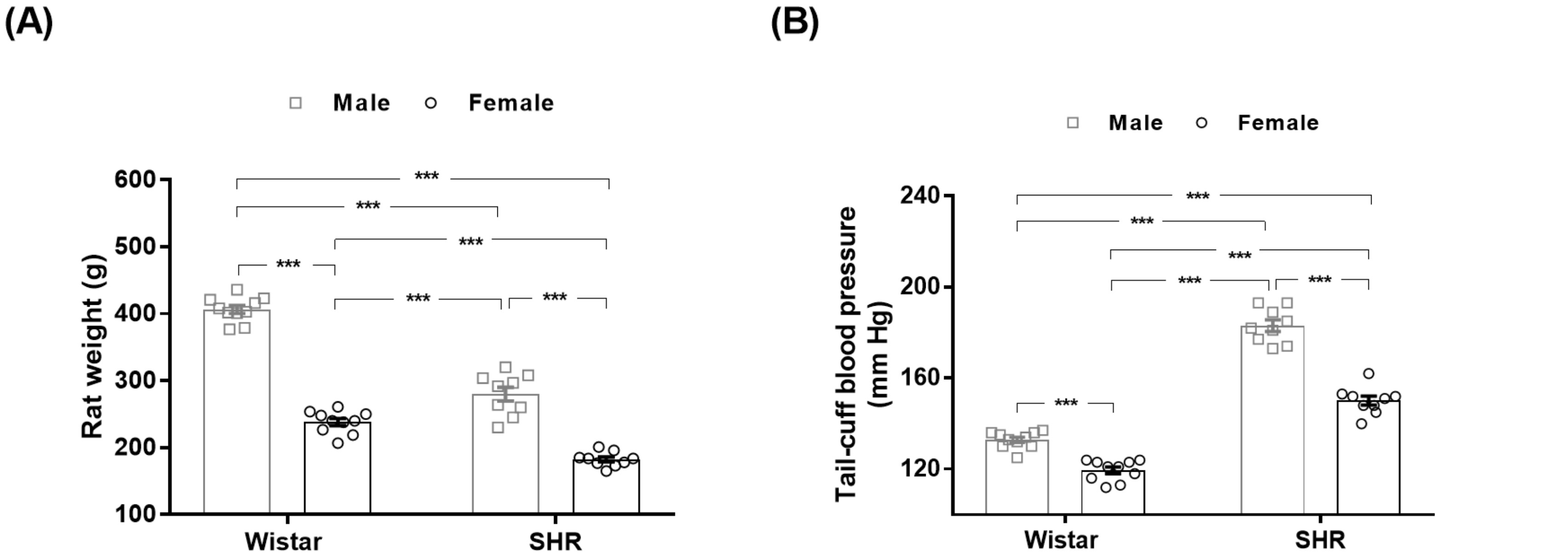

Figure S1. Rat weight and tail-cuff blood pressure of male and female normotensive (Wistar) and hypertensive (SHR) rats. (A) Rat weight and **(B)** tail-cuff blood pressure. Experimental n was indicated as scatter-dot, and the values represent individual measurements. Data were expressed as mean ± SEM, and ***P< 0.001 were based on a two-way ANOVA and 'Tukey's multiple comparisons post-test.

Figure S2

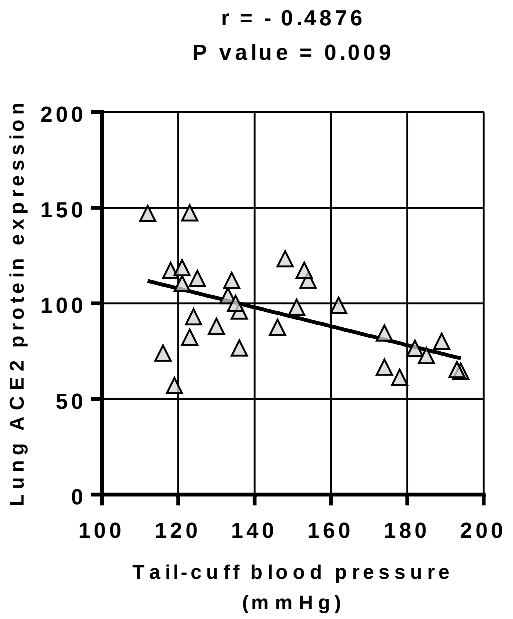

Figure S2. The relationship between lung ACE2 protein abundance and blood pressure. The correlation coefficient (*r*) and P-value were obtained by Pearson's correlation test, and lines were obtained by linear regression plotting.

Figure S3

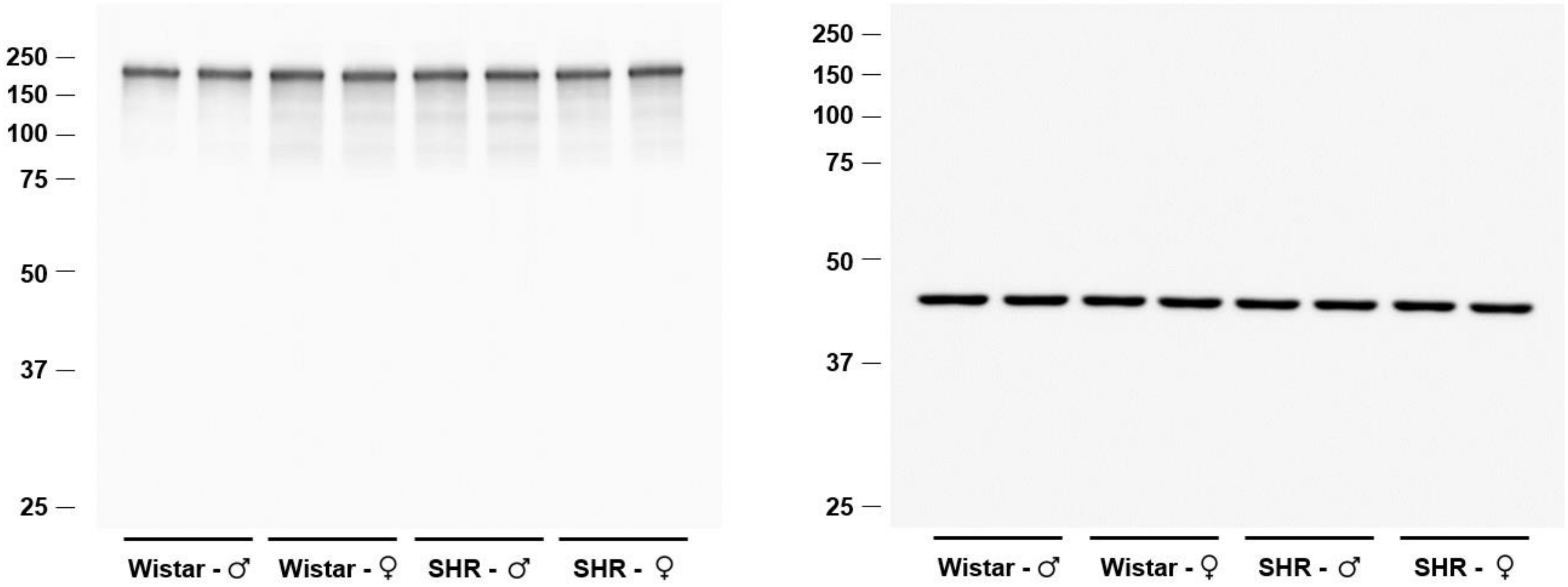

Figure S3 – Full unedited gel for Figure 1D. Left panel: ACE and Right panel: β-actin, in the lung of male and female normotensive (Wistar) and hypertensive (SHR) rats. All lanes have been used to build Figure 1D.

Figure S4

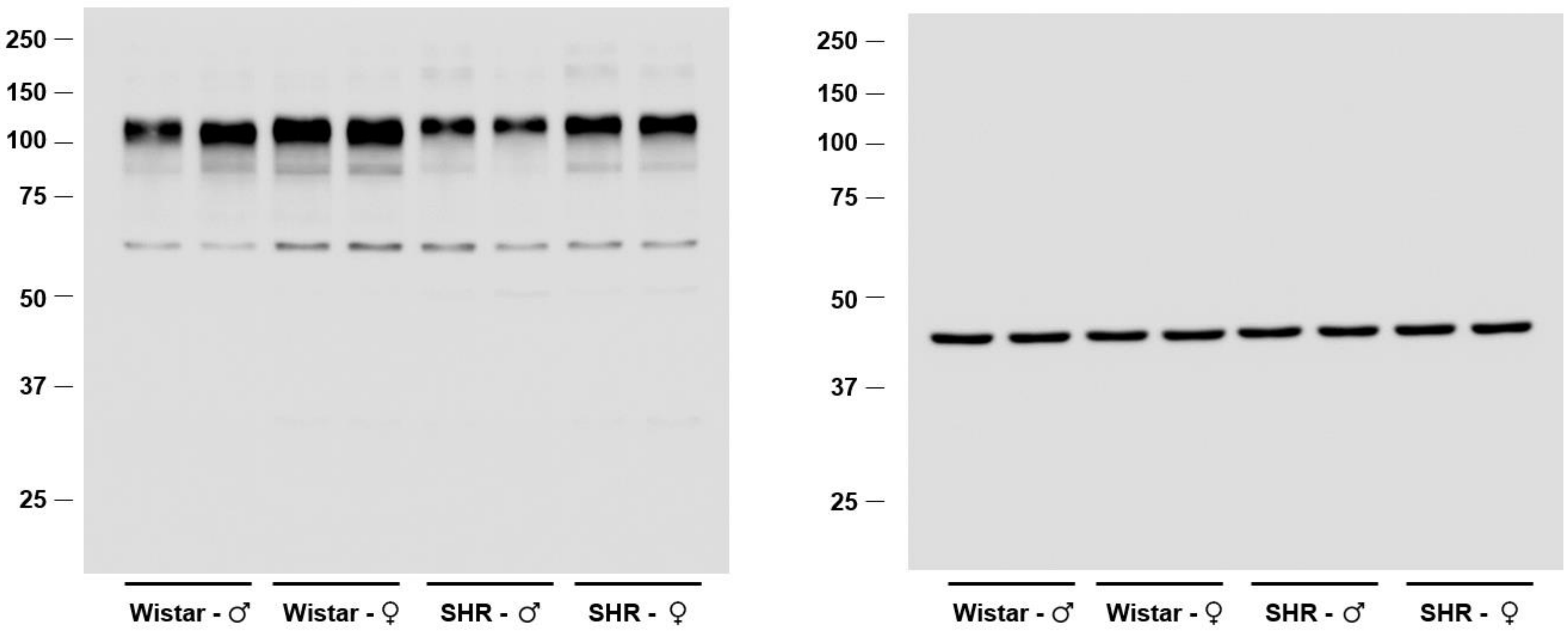

Figure S4 – Full unedited gel for Figure 1E. Left panel: ACE2 and Right panel: β-actin, in the lung of male and female normotensive (Wistar) and hypertensive (SHR) rats. All lanes have been used to build Figure 1E.

Table S1 - Age and body mass index (BMI) of individuals present in the GTEx portal database and used for the analysis of pulmonary gene expression of ACE and ACE2

		Normotensive	Hypertensive
n		311	74
**Male**		217	56
**Female**		94	18
**Age**	*Average*	55.4	58.7
	SD	12.2	8.0
	Max	70.0	70.0
	Min	21.0	34.0
	Median	57.0	59.0
**BMI**	*Average*	27.7	28.3
	SD	4.2	3.8
	Max	35.0	34.9
	Min	17.0	19.0
	Median	27.9	28.3
